# Supplementary figures and images for: A metagenomic comparison of clearwater, probiotic, and Rapid BFTTM on Pacific whiteleg shrimp, Litopenaeus vannamei cultures
Source: PeerJ. 2023 Sep 28;11:e15758. doi: 10.7717/peerj.15758 (PMC10542392; doi:10.7717/peerj.15758)

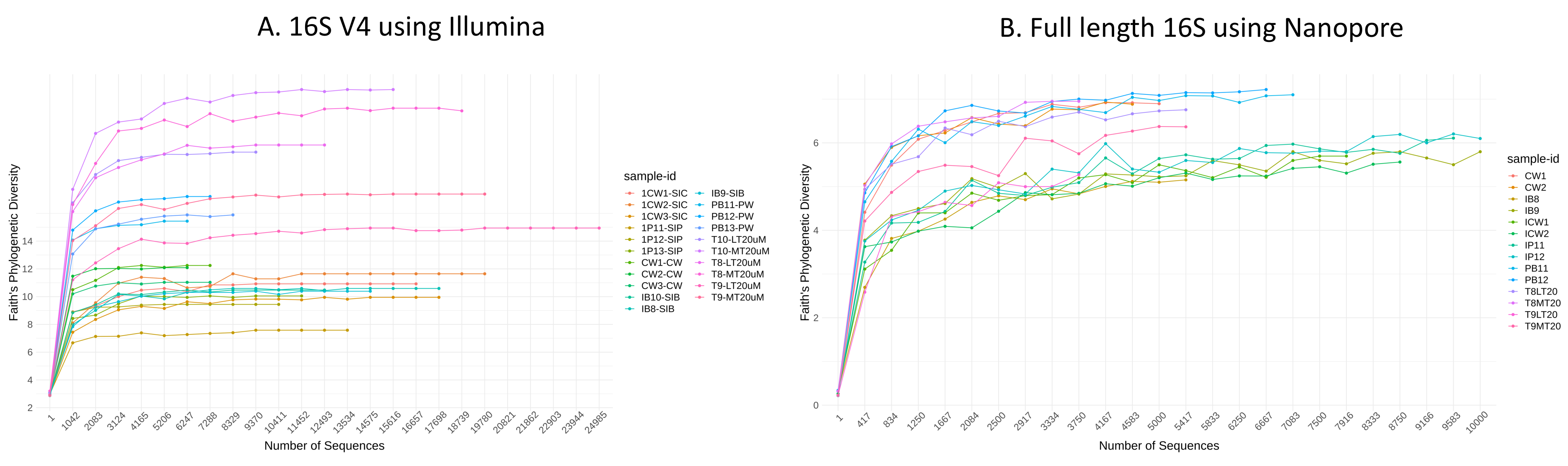

Supplement: Supplemental Information 4 — (A) shrimp guts reared in clear water (1CW1-SIC, 1CW2-SIC, 1CW3-SIC), probiotic (1P11-SIP, 1P12-SIP, 1P13-SIP), and biofloc (IB8-SIB, IB9-SIB, IB10-SIB); water samples from clear water (CW1-CW, CW2-CW, CW3-CW), probiotic (PB11-PW, PB12-PW, PB13-PW), biofloc of <20 μm (T8-LT20 uM, T9-LT20 uM, T10-LT20 uM), and biofloc >20 μm (T8-MT20 uM, T9-MT20 uM, T10-MT20 uM). (B) shrimp guts reared in clear water (ICW1, ICW2), probiotic (IP11, IP12), and biofloc (IB8, IB9); water samples from clear water (CW1, CW2), probiotic (PB11, PB12), biofloc of <20 μm (T8LT20, T9LT20), and biofloc >20 μm (T8MT20, T9MT20). [file peerj-11-15758-s004.png]
